# Rosettes integrity protects *Plasmodium vivax* of being phagocytized

**DOI:** 10.1038/s41598-020-73713-w

**Published:** 2020-10-07

**Authors:** Letusa Albrecht, Stefanie C. P. Lopes, Ana Beatriz Iung Enembreck da Silva, Vanessa Barbosa, Rodrigo P. Almeida, André M. Siqueira, Juliana Almeida Leite, Najara C. Bittencourt, Hellen Geremias dos Santos, Catarina Bourgard, Luiz Fernando Cardoso Garcia, Ana Carolina A. V. Kayano, Irene S. Soares, Bruce Russell, Laurent Rénia, Marcus V. G. Lacerda, Fabio T. M. Costa

**Affiliations:** 1 Laboratório de Pesquisa em Apicomplexa, Instituto Carlos Chagas, Fiocruz Paraná, Curitiba, PR Brazil; 2 Laboratório de Doenças Tropicais Prof. Luiz Jacintho da Silva, Departamento de Genética, Evolução, Microbiologia e Imunologia, Instituto de Biologia, Universidade Estadual de Campinas (UNICAMP), Campinas, SP Brazil; 3 Fundação de Medicina Tropical Dr. Heitor Vieira Dourado, Gerência de Malária, Manaus, AM Brazil; 4 Instituto Leônidas & Maria Deane, Fiocruz Amazônia, Manaus, AM Brazil; 5 Instituto Nacional de Infectologia Evandro Chagas, Fiocruz, Rio de Janeiro, RJ Brazil; 6 Department of Clinical and Toxicological Analyses, School of Pharmaceutical Sciences, University of Sao Paulo, São Paulo, SP Brazil; 7 Department of Microbiology and Immunology, University of Otago, Dunedin, New Zealand; 8 Singapore Immunology Network, Agency for Science, Technology and Research, Singapore, Singapore

**Keywords:** Malaria, Parasite immune evasion

## Abstract

*Plasmodium vivax* is the most prevalent cause of malaria outside of Africa. *P. vivax* biology and pathogenesis are still poorly understood. The role of one highly occurring phenotype in particular where infected reticulocytes cytoadhere to noninfected normocytes, forming rosettes, remains unknown. Here, using a range of ex vivo approaches, we showed that *P. vivax* rosetting rates were enhanced by plasma of infected patients and that total immunoglobulin M levels correlated with rosetting frequency. Moreover, rosetting rates were also correlated with parasitemia, IL-6 and IL-10 levels in infected patients. Transcriptomic analysis of peripheral leukocytes from *P. vivax*-infected patients with low or moderated rosetting rates identified differentially expressed genes related to human host phagocytosis pathway. In addition, phagocytosis assay showed that rosetting parasites were less phagocyted. Collectively, these results showed that rosette formation plays a role in host immune response by hampering leukocyte phagocytosis. Thus, these findings suggest that rosetting could be an effective *P. vivax* immune evasion strategy.

## Introduction

Outside of sub-Saharan Africa, malaria is mostly caused by *Plasmodium vivax*^1^. Although the pathogenesis of malaria is poorly understood, there is an increasing realization that this parasite has significant morbidity^2^.

Alterations in the dynamics of *Plasmodium* spp. infected red blood cells (iRBCs) due to their particular adhesiveness and deformability properties, are important contributors to malaria pathology^3^. While it is known that *P. vivax* alters the deformability of iRBCs^4^, its cytoadhesiveness to host endothelial cells has only recently been demonstrated^5, 6^.

In addition to the adhesiveness to endothelial cells, *P. vivax* iRBCs can also bind to noninfected red blood cells (RBCs), forming rosettes. Traditionally, malaria parasites are observed in a blood smear which difficult the observation of rosettes, since those could be disrupted by the mechanical force. Although rosetting in vivax malaria has been identified for more than two decades^7^, its role is still poorly understood. Contrasting with *P. falciparum* infections, in which rosetting has been associated with severity^8^, no association with clinical complications have been reported for vivax malaria^9^. *P. vivax* iRBCs specific cytoadhere to normocytes (rather than reticulocytes) through the glycophorin C receptor^9^. Interestingly, the adhesive force between the *P. vivax* iRBCs and noninfected erythrocytes is as strong as those observed in *P. falciparum*^10^. Clearly, rosetting is an important adaptation, but its specific role and the mechanisms behind the formation of *P. vivax* rosettes are unknown.

In the present study, sets of freshly *P. vivax* isolates were collected from Manaus, Brazil, and a variety of controlled ex vivo experiments were performed to mechanistically study host and parasite factors that may contribute to *P. vivax* rosetting.

## Results

### *Plasmodium vivax* rosettes are enhanced by plasma from infected patients

**Figure 1:**
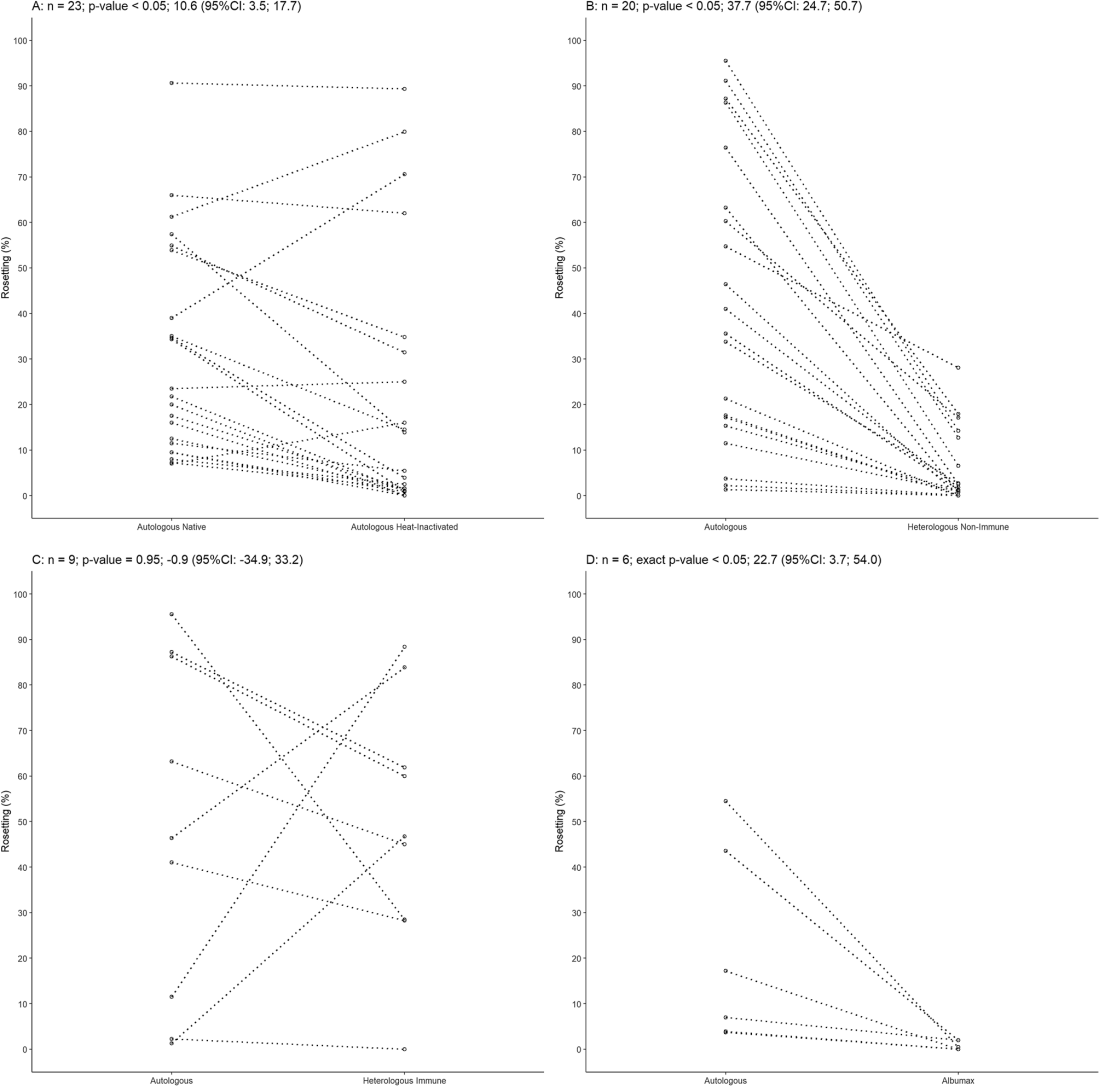
Features of *Plasmodium vivax* rosettes. (**A**) Rosetting in the presence of autologous native or heat-inactivated plasma (Student’s t-test, *p* < *0.05*, n = 23). (**B**) Rosetting of *P. vivax* in the presence of heterologous nonimmune or autologous plasma. Rosetting was eightfold higher in the presence of autologous plasma (Student’s t-test, *p* < 0.05, n = 20). (**C**) Rosetting of *P. vivax* in the presence of heterologous plasma from a high rosetting isolate (heterologous immune plasma). No difference in rosetting was observed between autologous and heterologous immune plasma (Student’s t-test, *p* = 0.95, n = 9) (**D)** Rosettes did not form on medium containing Albumax II (Wilcoxon test, *p* < 0.05, n = 6). CI: confidence interval.

Rosetting was assessed in a total of 81 *P. vivax* isolates and all samples formed rosettes at some extended in presence of autologous plasma. Rosettes were evaluated at late stages (late trophozoites/early schizont stages, 30–44 h post infection), and a mean rosetting of 38.78% ± 27.66 per isolate was found with a range from 1.30 to 95.51% (Supplementary Fig. S1). Rosettes were also evaluated in presence of native autologous plasma and heat-inactivated autologous plasma and it was found to be higher in the first condition (Student’s t test, *p* < 0.05, n = 23) (Fig. 1A). When comparing rosetting in the presence of autologous or a nonimmune heterologous plasma from a donor without history of malaria and no detectable antibodies to PvAMA-1 or PvMSP-1, rosetting rates were higher in the first group (Fig. 1B). Few rosettes were observed in presence of heterologous nonimmune plasma ranging from non-rosettes to 28.1% rosetting (Fig. 1B). Surprisingly, when these isolates were evaluated in the presence of autologous plasma, rosetting was enhanced by eightfold (Student’s t-test, *p* < 0.05, n = 20) (Fig. 1B). However, no significant differences were observed when a subset of isolates were assessed for rosetting in the presence of a heterologous immune plasma from a highly rosetting isolate (rosetting rate of 91.1%) (Student’s t-test, *p* = 0.95, n = 9) (Fig. 1C). Rosettes were not formed in plasma free medium containing Albumax II (Wilcoxon test, *p* < 0.05, n = 6), indicating that plasma components are important (Fig. 1D). Hematological parameters as platelet levels and hematocrit were also evaluated and it was found to be independent of rosetting rates (Person correlation coefficient, Supplementary Fig. S2A,B).

### Rosetting correlates with total immunoglobulin M but not IgG in patients infected with *P. vivax*

**Figure 2:**
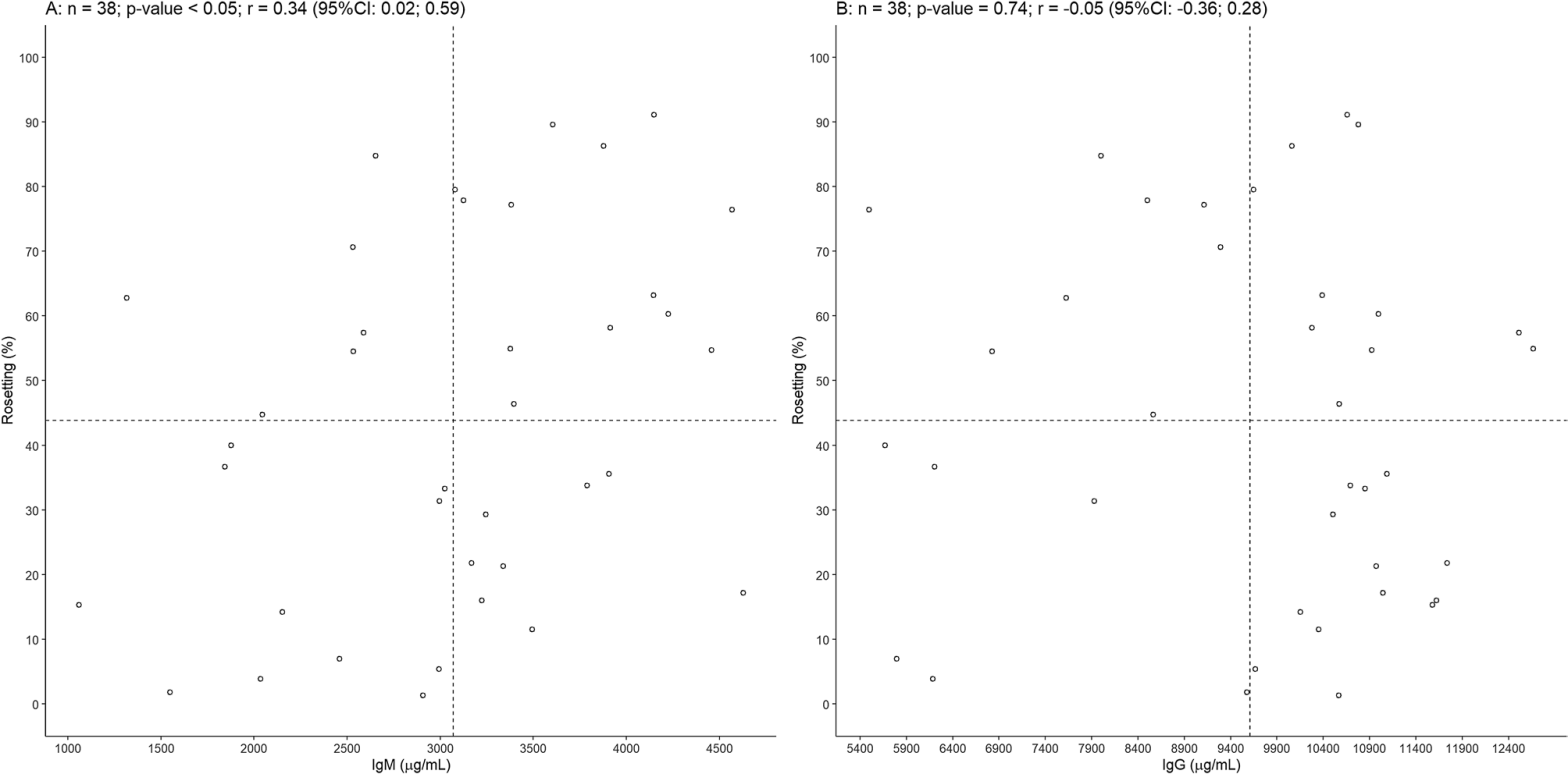
Total IgM but not IgG correlated with rosetting in vivax malaria. (**A**) Correlation of total IgM (Pearson correlation coefficient *r* = 0.34, *p* < 0.05, n = 38) and (**B**) total IgG (Pearson correlation coefficient *r* = − 0.05, *p* = 0.74, n = 38) from malaria infected patients and rosetting rate of *P. vivax* isolates. CI: confidence interval.

Immunoglobulins are among the plasma components that influence rosetting in *P. falciparum*^11^. Therefore, total immunoglobulin M (IgM) and G (IgG) were quantified in plasma from 38 *P. vivax* patients. IgM levels ranged from 1059 to 4628 μg/mL (mean = 3070 μg/mL), and IgG varied from 5498 to 12,669 μg/mL (mean = 90,608 μg/mL). Rosetting was positively correlated with IgM levels (Pearson correlation coefficient *r* = 0.34, *p* < 0.05) (Fig. 2A), but not with IgG levels (Pearson correlation coefficient *r* = − 0.05, *p* = 0.74) (Fig. 2B). The ability of IgM and IgG in *P. vivax*-infected patients to bind to noninfected RBCs was next tested. However, neither the binding of IgM or IgG to noninfected RBC correlated with rosetting rates in *P. vivax* patients (Supplementary Fig. S3).

### Naturally acquired antibodies to merozoite antigens and rosetting in vivax malaria

**Figure 3:**
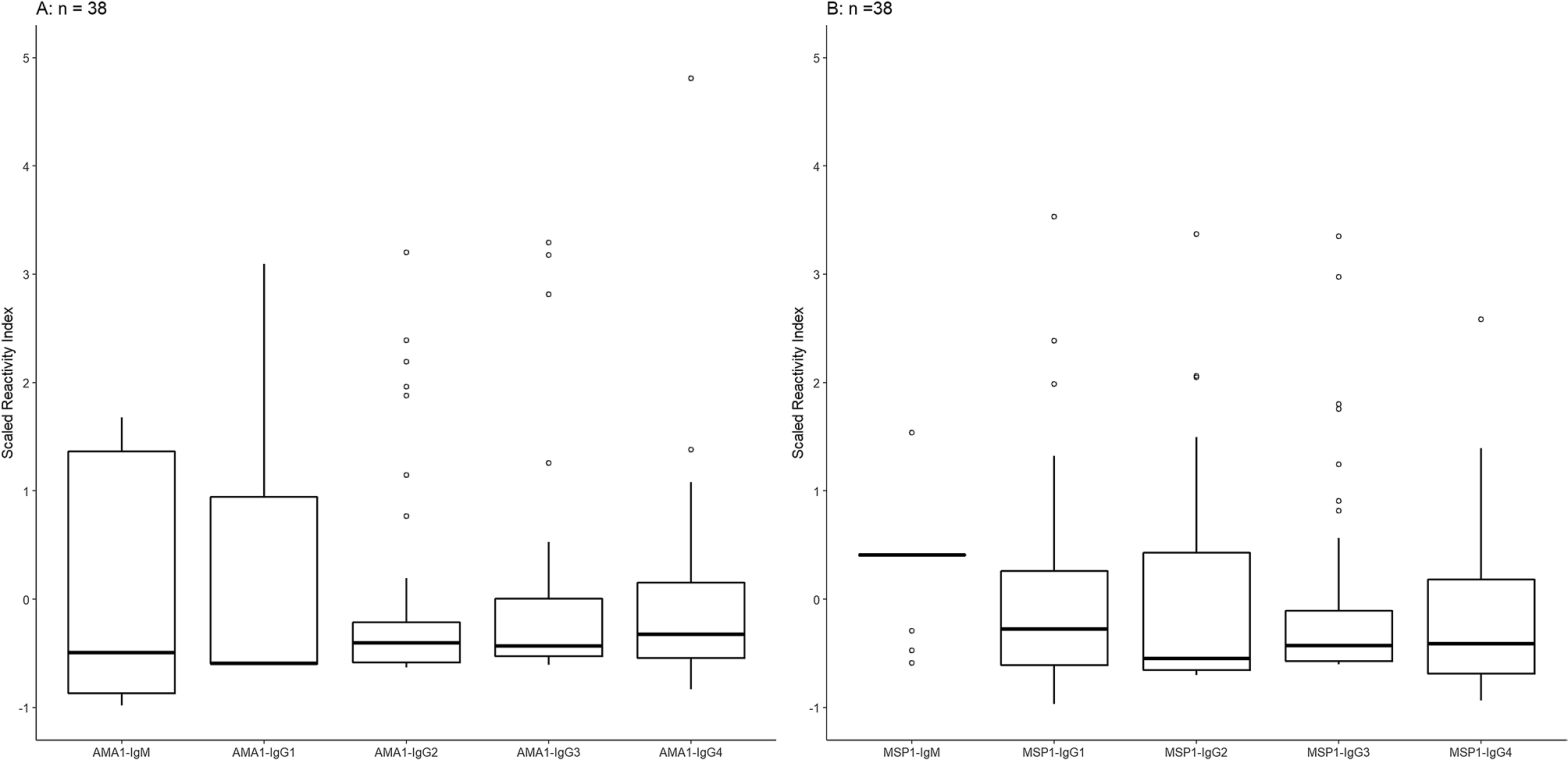
Naturally acquired IgG and IgM antibodies to merozoite antigens and its relationship with rosetting. (**A**) Boxplot showing median and interquartile ranges of scaled reactivity index for IgG subclasses (IgG1, IgG2, IgG3 and IgG4) and IgM towards PvAMA-1 and (**B**) PvMSP-1 recombinant proteins. Open circles denote outliers observations.

Because rosetting was correlated with total IgM, the relationship of specific *P. vivax*-related antibodies with rosetting was investigated. The presence of antibodies against two merozoite proteins, PvAMA-1 (Fig. 3A, n = 38) and PvMSP-1 (Fig. 3B, n = 38), was investigated. Most individuals had IgG1 antibodies to both proteins (57.9%, n = 22). The presence of anti-PvAMA-1 IgG1 antibodies was detected in 57.9% (n = 22) of individuals; 21.1% (n = 8) had IgG3; 10.5% (n = 4) had IgG4 and 7.9% had IgG2 (n = 3). The mean of rosetting was 46.97% in *P. vivax* isolates from individuals that had IgG1 antibodies to PvAMA-1 and 39.62% in *P. vivax* isolates from individuals with no detectable IgG1 antibodies. The mean of rosetting in *P. vivax* isolates from individuals with IgG3 antibodies to PvAMA-1 was 50.23% while in the group with no detectable IgG3 antibodies was 42.18%. Regarding the presence of antibodies to PvMSP-1, most samples had IgG1 and IgG3 antibodies, whereas 84.21% (n = 32) of samples were detected with IgG1; 86.84% (n = 33) with IgG3; 31.6% (n = 12) with IgG2 and 73.68% (n = 28) with IgG4. Infected individuals with detectable IgG1 antibodies to PvMSP-1 had a mean of rosetting of 44.22% while individuals whithout IgG1 antibodies had *P. vivax* rosettes with a mean of 42.04%. The mean of *P. vivax* rosetting was 45.74% in individuals with IgG3 against PvMSP-1 and 31.62% in individuals with no detectable antibodies.

### Parasitemia, IL-6 and IL-10 are increased in patients with high frequency of *P. vivax* rosettes

**Figure 4:**
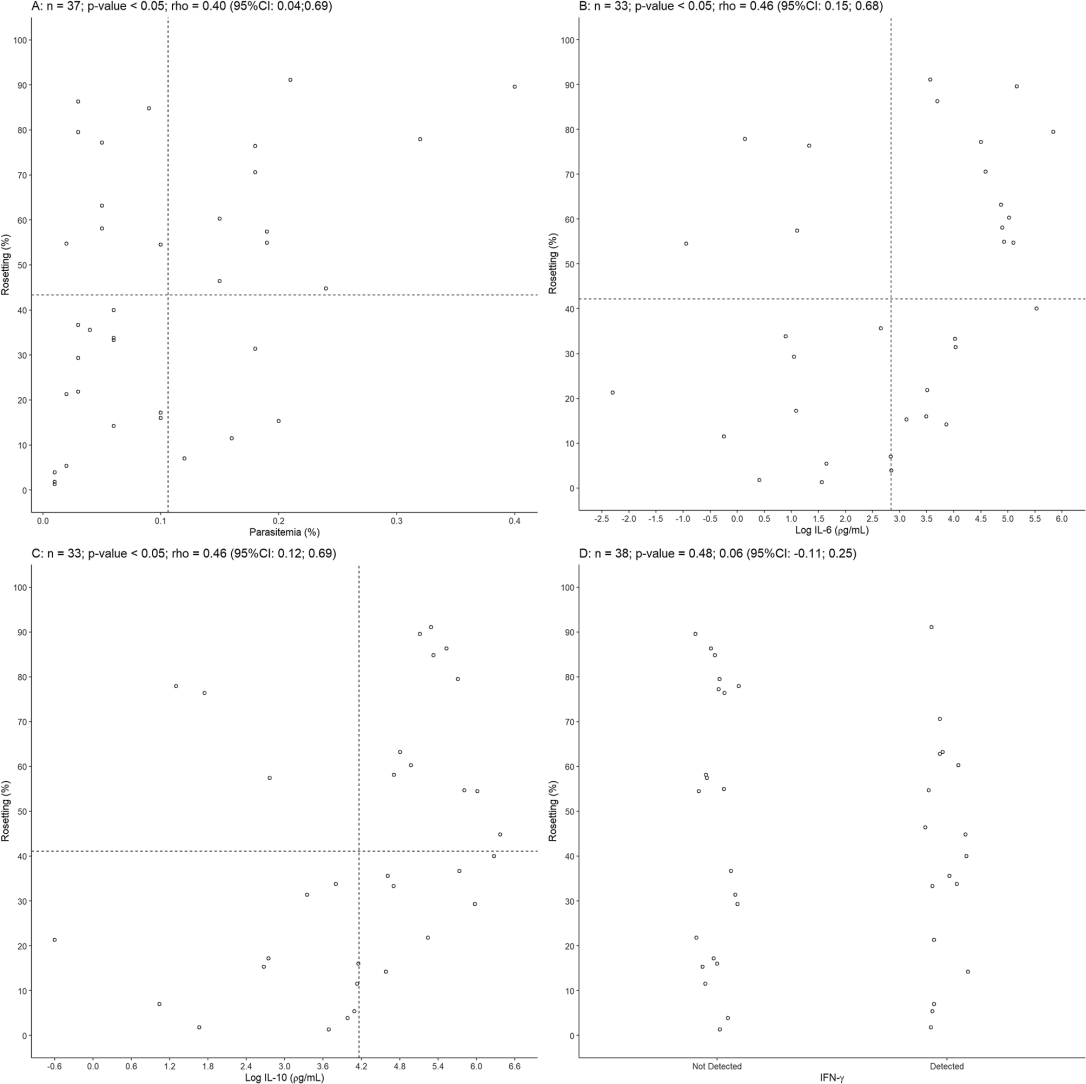
Parasitemia and plasmatic levels of IL-6 and IL-10 correlated with rosetting in vivax malaria. (**A**) Positive correlation between peripheral parasitemia and rosetting (Spearman’s correlation coefficient *rho* = 0.40, *p* =  < 0.05, n = 37). (**B**) Correlation of *P. vivax* rosetting and log IL-6 (Spearman’s correlation coefficient *rho* = 0.46, *p* < 0.05, n = 33). (**C**) log IL-10 (Spearman’s correlation coefficient *rho* = 0. 46, *p* < 0.05 and n = 33) and (**D**) IFN-γ (Student’s t-test, *p* = 0.48 and n = 38). CI: confidence interval.

Other parameter evaluated among studied isolates was parasitemia. When rosetting rate was compared with parasitemia a positive correlation was found (Spearman’s correlation coefficient *rho* = 0.40, *p* < 0.05, n = 37) (Fig. 4A).

Parasitemia has being associated with increased levels of specific cytokines in vivax malaria^12^. Considering that rosetting levels were associated with parasitemia the role of plasmatic cytokines in rosetting was investigated. Plasmatic cytokine IFN-γ, IL-6 and IL-10 levels were elevated in infected patients compared to noninfected individuals. Concentrations of IL-6, IL-10 and IFN-γ varied from 0.1 to 10,489.0 ρg/mL, 0 to 9416.75 ρg/mL and 0 to 26.8 ρg/mL, respectively. Five and four individuals had atypical values for IL-6 and IL-10, respectively, and were considered outliers, and one individual had no detectable value for IL-10 and therefore disregarded in further analysis. The logarithm of IL-6 and IL-10 were positively correlated with rosetting rates (IL-6, Spearman’s correlation coefficient *rho* = 0.46, *p* < 0.05 and n = 33; IL-10, Spearman’s correlation coefficient *rho* = 0.46, *p* <  0.05 and n = 33)  (Fig. 4B,C) while IFN-γ level was not associated with rosetting (Student’s t-test, *p* = 0.48 and n = 38) (Fig. 4D). In addition, the logarithm of IL-6 and IL-10 were also positively correlated (Spearman’s correlation coefficient *rho* = 0.54, *p* < 0.05, n = 30) (Supplementary Fig. S4A). However, no association were found between cytokines and parasitemia (Supplementary Fig. S4B–D).

### Genes related to phagocytosis pathway are differentially expressed in peripheral blood mononuclear cells of patients infected with *P. vivax *forming rosettes

**Figure 5:**
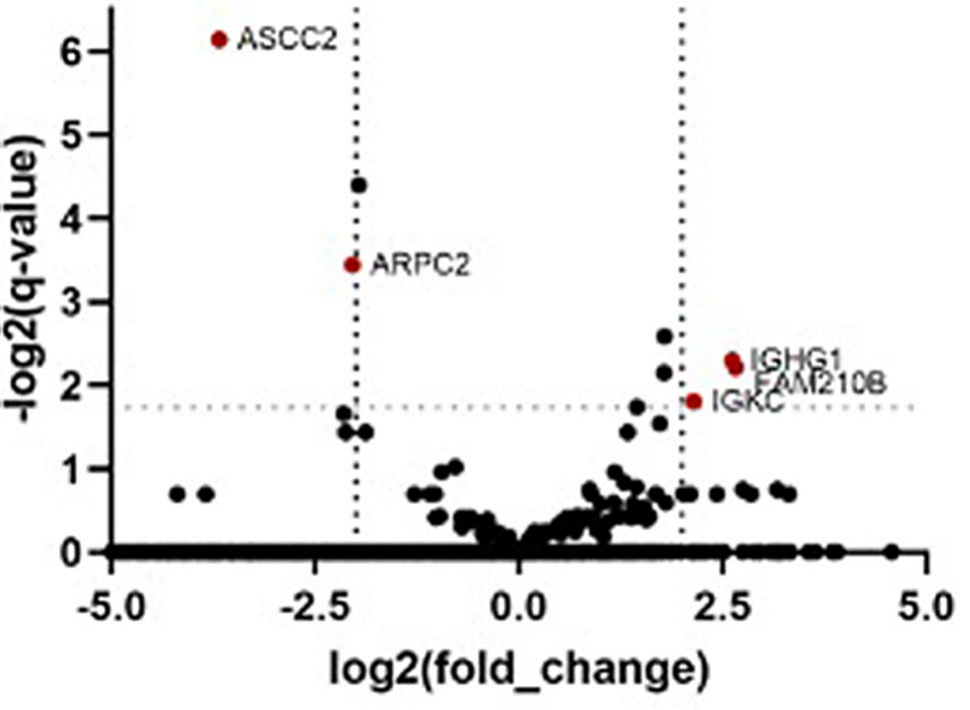
RNAseq analysis of PBMCs from patients infected with *P. vivax*. Volcano plot showing the range of the log2(fold change) relative to the − log2(q-values) of mapped genes from PBMCs of *P. vivax* infected patients between moderate and low rosetting isolates differential expression data analysis. Identified genes with a p-value < 0.05, q-value < 0.3 and log2 (fold change) > 2 cut-offs obtained from RNAseq differential gene expression analysis are put in evidence (dark red dots).

Transcriptome analysis was performed on peripheral leukocytes from *P. vivax* infected patients with very low or moderate rosetting (Table 1). Five genes that showed a log2 fold change higher than 2 were identified (Table 2, Fig. 5). Three out of five genes were in the Fc gamma receptor (FCGR)-dependent phagocytosis pathway. Immunoglobulin kappa constant (IGKC) and immunoglobulin heavy constant gamma 1 (IGHG1) were upregulated and actin-related protein 2/3 complex subunit 2 (ARPC2) was downregulated in individuals with moderate rosetting compared to patients with low rosetting.

**Table 1:** Summarized description of patients infected with *P. vivax* analyzed by RNAseq.

Group analysis	Sample code	Rosette formation (%)
Moderate rosetting	106U16	30.0
	69U15	24.4
Very low rosetting	109U16	6.0
	73U15	5.6

**Table 2:** RNAseq analysis of PBMCs from patients infected with *P. vivax*.

Gene ID	Gene name	Gene description	Gene locus	Log2 (fold change)	q-value
ENSG00000100325.14	ASCC2	Activating Signal Cointegrator 1 Complex Subunit 2	chr22:29788607–29838304	− 3.67	0.01
ENSG00000163466.15	ARPC2	Actin Related Protein 2/3 Complex Subunit 2	chr2:218217093–218254356	− 2.04	0.09
ENSG00000211592.7	IGKC	Immunoglobulin Kappa Constant	chr2:88811185–89245596	2.14	0.29
ENSG00000211896.7	IGHG1	Immunoglobulin Heavy Constant Gamma 1	chr14:105664632–106538344	2.62	0.20
ENSG00000124098.9	FAM210B	Family with Sequence Similarity 210 Member B	chr20:56358914–56368663	2.66	0.22

Graph of log2 fold change of genes from PBMC of *P. vivax* infected patients between moderate and low rosetting isolates data analysis. Gene lists using p-value < 0.05, q-value < 0.3 and log2 (fold change) > 2 cut-offs obtained from RNAseq differential gene expression analysis.

### Rosetting protects *P. vivax* parasites from phagocytosis

**Figure 6:**
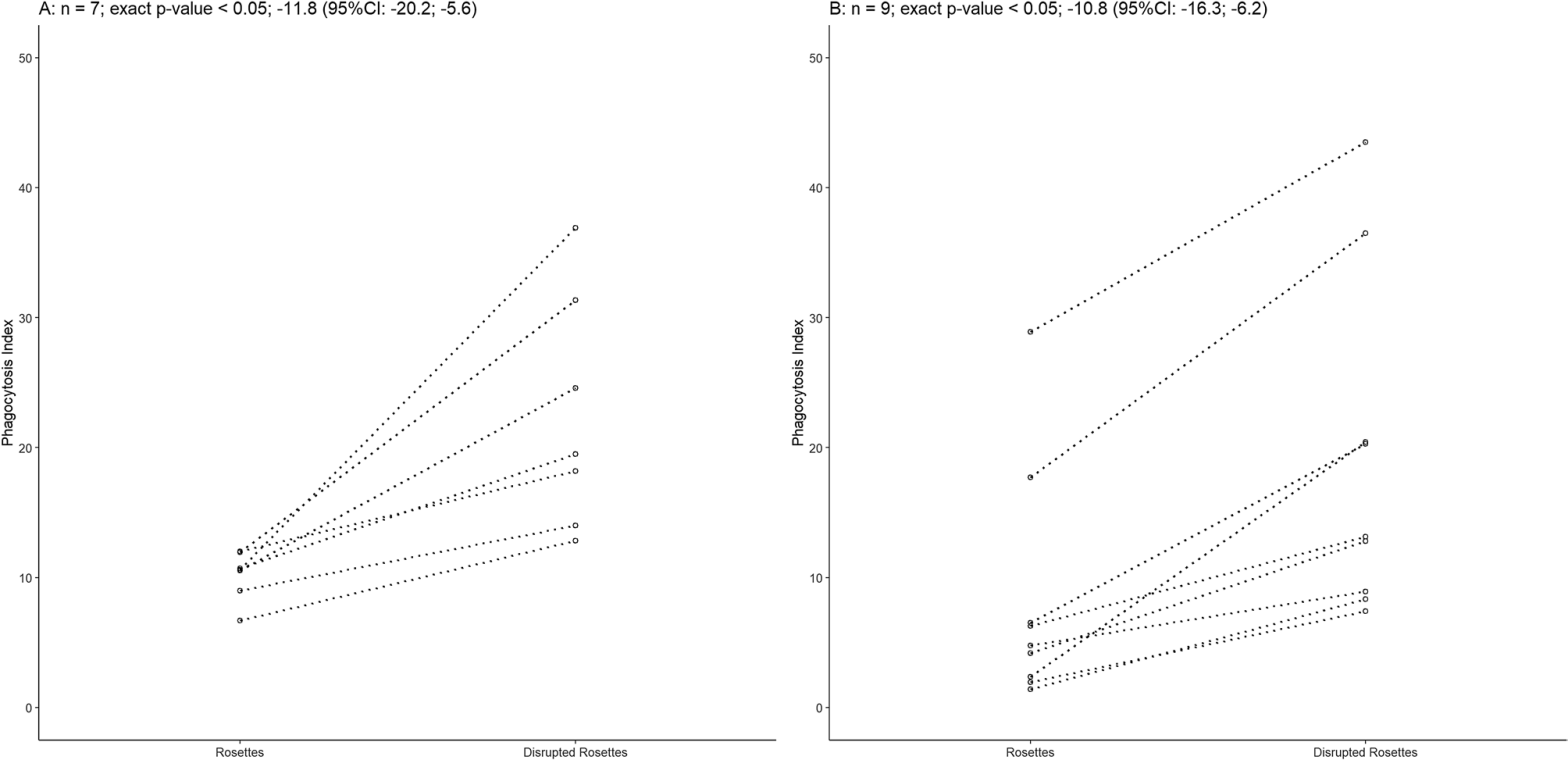
Non-rosetting parasites are more likely to be phagocyted. (**A**) Rosetting-disrupted *P. vivax* parasites were preferentially phagocyted in vitro by THP-1 cells (Wilcoxon test, *p* = 0.02, n = 7) as well as for **(B)** patients peripheral blood mononuclear cells (Wilcoxon test, *p* = 0.004, n = 9). CI: confidence interval.

Because transcriptional analysis indicated that a phagocytosis pathway may be affected by rosetting, functional assays were conducted to investigate the role of this adhesive phenomenon. Phagocytosis assay was performed using THP-1 human monocytic cell line and *P. vivax* rosette isolates at two different conditions: intact or mechanically disrupted. For THP-1 cells the higher phagocytosis index was observed when rosettes were mechanically disrupted. The mean difference of phagocytosis index was − 11.8 (95% confidence interval (CI) − 20.2; − 5.6) (Wilcoxon test, *p* < 0.05, n = 7), (Fig. 6A). To confirm these results, phagocytosis assays were also performed with peripheral blood mononuclear cells (PBMCs) isolated from individuals infected with *P. vivax*. As observed to THP-1, phagocytosis index for PBMC were also higher when rosettes were mechanically disrupted. The mean difference of phagocytosis index was − 10.8 (95% CI − 16.3; − 6.2) (Wilcoxon test, *p* < 0.05, n = 9) (Fig. 6B).

## Discussion

Although it has been more than two decades that rosetting in *P. vivax* was reported^7^, its role in parasite pathobiology remains unknown. The present study showed that *P. vivax* are less phagocyted when forming rosettes rather than when these rosettes are disrupted. The results suggest rosetting as an evasion mechanism.

The ability of all *P. vivax* isolates to form rosettes at late stages suggested an important role of this adhesion phenomenon. Rosetting was revealed to be a gain for the parasite as confirmed by the positive correlation between rosetting and parasitemia. While the data presented here suggest that rosetting provides a biological advantage to *P. vivax*, the nature of this mechanism is not completely elucidated.

Rosettes does not facilitate merozoite invasion since it was showed by Lee et al. that *P. vivax* rosettes are preferentially formed by mature red blood cells which are refractory to *P. vivax* merozoite invasion^9^. It has also been speculated that rosetting may protects *P. vivax* from being eliminated by the host immune system, however no data has been provided to support this hypothesis^9,13^. Here, experimental data supported this latter idea that *P. vivax* uses rosetting to avoid a key aspect of the host immune response.

In malaria caused by *P. falciparum,* the rosetting success is highly dependent on plasma components^11,14^. In the present study, *P. vivax* parasites did not form rosettes in the presence of Albumax II and rosetting rate was eight times higher when autologous plasma was present. Rosetting rates were similar when in presence of autologous plasma or when in presence of a heterologous immune plasma, from an infected donor with high rosetting isolate, suggesting that a component, yet to be identified, in infected patients could favour *P. vivax* rosetting. IgM levels in plasma from *P. vivax* infected patients correlated with rosetting frequency. In falciparum malaria, some but not all rosetting strains are dependent on IgM^11^. A potential role of IgM in falciparum rosetting is to stabilize rosettes, acting as a bridge between infected and noninfected cells^15^. IgM may also assist *P. falciparum* in the host immune evasion as natural IgM binding to iRBCs have been shown to mask IgG epitopes^16^, thus limiting opsonization and phagocytosis. Although the present data suggested that IgM is also important in *P. vivax* rosettes the role of this immunoglobulin in adhesion phenotypes are not elucidated in vivax malaria. Specific IgM against PvAMA-1 and PvMSP-1 antibodies were not associated to rosetting. The same association was not observed for total IgG.

Antibodies to merozoite proteins are frequently detected in *P. vivax* infected patients and could be an indicator of previous malaria infection^17,18^. PvAMA-1 antibodies have being linked with malaria recent infection^19^ while MSP-1 antibodies might be an indicative of previous malaria exposition^20^. Most individuals here analyzed had IgG1 antibodies to PvMSP-1 and PvAMA-1 indicating that are likely that those individuals had a previous malaria episode.

Here, the correlation between hematocrit and rosetting was not observed. *P. vivax* rosetting has been previously associated with anemia during pregnancy^21^. Even though the mechanisms that are driving anemia in vivax malaria are not completely elucidate, there are evidences that the presence of autoantibodies are increased during acute *P. vivax* infection^22–25^.

A cytokine storm is frequently observed in vivax malaria which is a result of an excessive activation of innate immune cells^26^. Elevated levels of pro-inflammatory cytokines have been reported in acute *P. vivax* infection^27,28^. Besides that, the anti-inflamatory cytokine IL-10 has been also detected at high concentration in symptomatic malaria^29,30^. In this current study, rosetting was correlated with IL-6 and IL-10 levels, however the mechanism behind cytokine-aided *P. vivax* rosetting remains unknown. One of the attributes of IL-6 is to promote early production of parasite-specific IgM antibodies^31^. Here, no direct association between IL-6 and IgM levels was detected, but both parameters were correlated with rosetting. IL-10 levels have been correlated with higher parasitemia in vivax malaria^32,33^, whereas asymptomatic patients have lower levels of IL-10^34^. No direct association between IL-10 and parasitemia was found. However, parasitemia and IL-10 correlated with rosetting frequency. IL-10 has also been associated with previous malaria infection^12,29^, which could indicate that rosetting is also more frequent in individuals that were previously infected.

The host transcriptomic profile directly compared patient isolates with low versus moderate rosetting, which were quantified immediately after blood sample collection, when most parasites are young^6^. Rosetting is mostly formed by mature parasites, this constituted an experimental limitation of the present study. Additionally, a low quantity of host sample input effectively transcribed resulted in few statistically significant pools of differentially expressed genes. Nevertheless, three out of five genes showing more than twofold expression change are annotated as part of Fc gamma phagocytic pathway. Two of the genes up regulated in the moderate rosetting group are related to antibody production. These results suggest that patients with higher rosetting are producing higher amounts of antibodies but as phagocytosis assays indicate, rosettes are protecting parasites of being phagocyted. Besides that, one of the genes differentially expressed was the actin related protein (ARPC2), which is important for the remodelling of macrophages membrane during phagocytosis^35^. This gene was down regulated in moderate rosetting isolates, indicating that phagocytosis might be compromised in these individuals.

Finally, parasites that were in a rosetting formation were less likely phagocytized. When rosettes were mechanically disrupted, rates of phagocytosis were increased more than twofolds. The most likely explanation for this observation is that the noninfected erythrocytes in the rosette formation shielded the iRBCs from antibodies and provided a physical barrier to restrict contact with phagocytes and other immune effector cells.

Taken together, these data suggest that vivax malaria rosetting is an evasion mechanism that allows the parasite to escape from the host immune system. The fact that rosetting is a frequent feature in *P. vivax* late stages indicates that this phenotype could be an advantage for the parasite by conferring significant protection from the host immune system. Therefore, the understanding of *P. vivax* rosettes may help in the development of new strategies for malaria control.

## Material and methods

### Ethics statement

All procedures, including protocols and written consent forms, were approved by the Ethics Review Board of *Fundação de Medicina Tropical Dr. Heitor Vieira Dourado* (FMT-HVD; Manaus, Amazonas State, Brazil) (process CAAE-0044.0.114.000-11). Patients were adults and joined at this study after providing written informed consent. All methods were performed in accordance with the relevant guidelines and regulations.

### Blood sample collection

Blood samples were obtained from malaria patients presenting at the Hospital of the Fundação de Medicina Tropical Dr, Heitor Vieira Dourado (Manaus, Amazonas State, Brazil). Prior to sample collection, patients granted informed consent. Blood samples were collected using BD Vacutainer tubes with sodium citrate anticoagulant. A thin blood smear was prepared from each sample to determine species of malaria parasites involved, parasitemia and the predominant erythrocytic stage of the parasite. Blood cell count was performed immediately after blood collection using a Sysmex KX21N (Sysmex Corporation-Roche, Japan). After collection of peripheral blood, patients received treatment following national guidelines with chloroquine plus primaquine.

### Parasite isolation and enrichment

Once microscopic diagnosis of uncomplicated vivax malaria was made and before the treatment was initiated, 8 mL of blood was collected into citrate-coated Vacutainer tubes (BD). Blood was immediately processed to obtain enriched Pv-iRBCs. Immediately after collection, the RBCs containing trophozoites and schizonts were separated from the younger forms on a 45% Percoll (GE Healthcare) gradient as previously described^5^.

### Rosetting assay

IRBCs (20 µL) at 2.5–5% parasitemia and 2.5–5% hematocrit were initially washed three times with McCoy’s 5A medium (400 g, 5 min., at room temperature). After that, iRBC were incubated for 40 min at 37 °C in rosetting medium (McCoy’s 5A medium supplemented with 20% of autologous plasma). Duplicated samples were stained with 45 µg/ml acridine orange and examined by direct light and fluorescence microscopy (Nikon Eclipse 50i, filter 96311 B-2E/C). Rosetting was assessed by counting 200 iRBCs, in duplicate. Slides were counted at diagonal vision, to balance for a possible irregular distribution of rosettes in the slide. A rosette was determined by the binding of two or more uninfected erythrocytes to an iRBC (Supplementary Fig. S5). To assess the involvement of plasma factors in the rosette formation, the plasma in rosetting medium was substituted for 0,5% of Albumax II. For heat-inactivated plasma, samples were heat inactivated for 30 min at 56 °C. Nonimmune heterologous plasma evaluated was from a donor without history of malaria and no detectable antibodies to PvAMA-1 or PvMSP-1 (reactivity index < 1).

### Measurements of plasma cytokines and IgG and IgM levels

The levels of IL-2, IL-4, IL-6, IL-10 TNF-α and IFN-γ were quantified in cryopreserved plasma samples using the Cytometric Bead Array system (CBA; BD Biosciences) following the manufacturer’s instructions. Samples were analyzed using a BD FACSCalibur (BD Biosciences, USA). Standard curves were derived from cytokine standards for each cytokine analyzed. Total IgG and IgM were measured from plasma using an ELISA quantitation kit (Bethyl Laboratories) following the manufacturer’s recommendations.

### IgG and IgM red blood cell binding

Briefly, erythrocytes (A+) at 0.8% hematocrit in RPMI medium were incubated with plasma from *P. vivax-*infected patients at 1:10 dilution for 1 h. After three washing steps with RPMI, erythrocytes were incubated for 30 min with anti-IgG or anti-IgM Alexa Fluor 488 conjugate (ThermoFisher Scientific). Erythrocytes were washed thrice and suspended in PBS solution, and 100.000 events were acquired using a BD FACSCanto II (BD Biosciences, USA). Flow cytometry results were analyzed using FlowJo software.

### Naturally acquired IgG antibody subclasses of antibodies to PvAMA-1 and PvMSP-1_19_ merozoite antigens

Specific IgG subclasses of antibodies (IgG1, IgG2, IgG3 and IgG4) to PvAMA-1 and PvMSP-1_19_ were detected in plasma by enzyme-linked immunosorbent assays (ELISAs). PvAMA-1 and PvMSP-1_19_ recombinant proteins were expressed and purified as previously described^36,37^. ELISAs were conducted as previously described^38^. The results for IgG subclasses were expressed as reactivity indices (RIs), which were calculated by dividing the mean optical density (OD) values of tested samples by the mean OD values plus three standard deviations of 20 non-exposed individuals living in nonendemic areas of malaria. RI values > 1 were considered positive.

### RNAseq of peripheral blood mononuclear cells

mRNA from PBMCs isolated from total blood of patients infected with *P. vivax* with high (n = 2) or low (n = 2) rosetting was analyzed by RNAseq. For this specific approach, moderate rosetting isolates were considered as 20% rosettes, and low rosetting isolates were considered as less than 10% rosettes. RNA extractions were performed a RNeasy Micro Kit (Qiagen) according to the manufacturer’s instructions. Before conducting Whole Transcriptome Shotgun Sequence (WTSS), RNA quality was accessed using an Agilent 2100 Bioanalyzer as well as Agilent RNA 6000 Pico Kit reagents and chips, and it was analyzed using 2100 Expert software according to the recommendations of Agilent Technologies. A SMART-Seq V4 Ultra Low Input RNA Kit was used for sequencing via Clontech’s patented Switching Mechanism at 5′ End of RNA Template (SMART) technology. cDNA quality, quantity and size range were evaluated using the BioAnalyzer platform from Agilent Technologies, Inc. using the Agilent High Sensitivity DNA Kit (cDNA, 5 to 500 pg/µL within a size range of 50 to 7000 bp) following the manufacturer’s instructions. Prior to generating the final library for Illumina sequencing, the Covaris AFA system was used for controlled cDNA shearing. cDNA output was then converted into sequencing templates suitable for cluster generation and high-throughput sequencing through the Low Input Library Prep v2 (Clontech Laboratories, Inc.; Takara Bio Company). Library quantification procedures were performed by qPCR using the Library Quantification Kit (Clontech Laboratories, Inc.; Takara Bio Company) and Agilent's High Sensitivity DNA Kit (Agilent Technologies, Inc.), and they were successfully completed before proceeding to the pool setup at a final concentration of 2 nM for direct sequencing. The library was sequenced on a HiSeq 2500 sequencer on Rapid Run mode with the HiSeq Rapid Cluster Kit v2 (100 × 100) Paired End and HiSeq Rapid SBS Kit v2 (200 cycles). The generated libraries were cluster amplified and sequenced on the Illumina platform using standard Illumina reagents and protocols for multiplexed libraries following the manufacturer’s loading recommendations. On average, approximately 474.553 paired end reads were obtained per sample from the 4 sample libraries sequenced. The RNAseq raw reads were checked for quality by running Fast Quality Control (FastQC—https://www.bioinformatics.babraham.ac.uk/projectY/fastqc/). The reads were then subjected to a RNAseq alignment v1.1.1 workflow from BaseSpace platform for cloud-based genomics analysis and storage, and they were integrated with Illumina sequencers. This workflow allowed trimming of Illumina adaptors, read mapping using TopHat 2 (Bowtie 2) aligner towards the *Homo sapiens* UCSC hg38 (RefSeq & Gencode gene annotations)^39^, and FPKM estimation of reference genes and transcripts using Cufflinks 2. Final assembly and analysis of differentially expressed reference transcripts were performed with Cuffdiff 2 within the Cufflinks Assembly & DE pipeline v2.1.0. Differential gene expression between moderate and low rosetting groups was identified after a pairwise Wilcoxon test was used to compare the transcriptional profiles with the following cutoffs: p-value < 0.05, q-value < 0.5 and a log2 fold change > 2.

### Phagocytosis assay with *P. vivax*

THP-1 cells were grown on 8-well culture slides. Maturation was induced by incubation with 60 ng/mL phorbol 12-myristate 13-acetate (PMA) (CalbiochemH, San Diego, CA) for 24 h at 37 °C. The supernatant was then removed, and cells were washed twice with cell culture medium. In parallel, parasites were kept at room temperature in static conditions (intact rosettes) or agitated using a vortex for 30 min after complete disruption of rosettes using a needle (disrupted rosettes), in the presence of autologous plasma. *P. vivax* isolates (1 × 10^6^) at 5–8% parasitemia were added to each well of THP-1 cells. After 30 min of incubation in 5% CO_2_ atmosphere at 37 °C, THP-1 cells were harvested and washed three times in cell culture medium, and the slides were fixed in methanol and stained with panoptic stain. At least 200 THP-1 cells were counted in each well, and all samples were tested in duplicated. THP-1 cell culture was checked for *Mycoplasma* contamination weekly.

Twenty mL of total blood were used to separate PBMC using Ficoll-Paque-PLUS following the fabricant instructions. After isolation, cells were counted at optical microscopy and 1 × 10^6^ cells were added to a Labtek slide and kept for 4 h at 37 °C. Non-adherent cells were removed and phagocytosis assay was performed. Immediately before phagocytosis assay, parasites collect from the same patients were kept at room temperature in static conditions (intact rosettes) or agitated using a vortex for 30 min after complete disruption of rosettes using a needle (disrupted rosettes), in the presence of autologous plasma. *P. vivax* isolates (2 × 10^6^) at 5–8% parasitemia were added to each slide of PBMC. After 1h30min of incubation at 37 °C, PBMCs were harvested and washed three times in cell culture medium, and the slides were fixed in methanol and stained with panoptic stain. At least 200 PBMCs were counted in each well, and all samples were tested in duplicated.

### Statistics

Data were analyzed using R software version 3.6.2. For quantitative variables, we analyzed the frequency distribution, measures of central tendency (mean or median) and variability (standard deviation or interquartile ranges), and searched for outlier observations (those values above [75 percentile value + 1.5 * IQR] or below [25 percentile value – 1.5 * IQR], IQR: interquartile range, represented by percentile 75 value minus percentile 25 value). Additionally, for IL-6 and IL-10 variables, we applied logarithm transformation to stabilize its high variability and for IFN-γ we have dichotomized its values in not detect (0 values) and detected (above 0 values) IFN-γ. For immunoglobulin reactivity indexes joint distribution presentation, we have scaled these variables (subtraction of the mean and division by standard deviation) since it mean and standard deviation were markedly different.

In bivariate analysis, for paired data comparisons of rosetting rate between autologous plasma and the following conditions—heat-inactivated plasma, heterologous nonimmune plasma, heterologous immune plasma and Albumax—and for comparisons of phagocytose index between integrated and disrupted rosette groups we applied paired Student t-test or Wilcoxon test, depending on the distribution of the difference of the variable of interest between paired observation. For comparisons of rosetting rate between detected and not detected IFN-γ groups, we applied t-test. To investigate possible relationships between quantitative variables, we used Pearson (r) in case distribution approximately normal, and Spearman’s rank correlation coefficient (rho) otherwise, with correspondent 95% confidence intervals.

A *p*-value < 0.05 was considered to be statistically significant. All statistical tests applied are indicated in figures caption, as well as the number of samples analyzed.

## Supplementary information

Supplementary Information.

## Data Availability

All data generated or analyzed during this study are included in this published article (and its Supplementary Information files). Deep sequencing data was deposit in Array Express, accession number E-MTAB-8385.
