# Supplementary material for: Rosettes integrity protects Plasmodium vivax of being phagocytized
Source: Sci Rep. 2020 Oct 7;10:16706. doi: 10.1038/s41598-020-73713-w (PMC7541459; doi:10.1038/s41598-020-73713-w)

Supplementary Information

Title: Rosettes integrity protects *Plasmodium vivax* of being phagocytized

Letusa Albrecht [1,2,*], Stefanie C.P. Lopes[3,4], Ana Beatriz Iung Enembreck da Silva[1], Vanessa Barbosa[3,4], Rodrigo P. Almeida[1], André M. Siqueira[5], Juliana Almeida Leite[2], Najara C. Bittencourt[2], Hellen Geremias dos Santos[1], Catarina Bourgard[2], Luiz Fernando Cardoso Garcia[1], Ana Carolina A. V. Kayano[1,2], Irene S. Soares[6], Bruce Russell[7], Laurent Rénia[8], Marcus V. G. Lacerda [3,4] and Fabio T.M. Costa[2*]

[1]Laboratório de Pesquisa em Apicomplexa, Instituto Carlos Chagas, Fiocruz Paraná, Curitiba, PR, Brazil.

[2]Laboratório de Doenças Tropicais, Departamento de Genética, Evolução, Microbiologia e Imunologia, Instituto de Biologia, Universidade Estadual de Campinas (UNICAMP), Campinas, SP, Brazil.

[3]Fundação de Medicina Tropical Dr. Heitor Vieira Dourado, Gerência de Malária, Manaus, AM, Brazil.

[4] Instituto Leônidas & Maria Deane, Fiocruz Amazônia, Manaus, AM, Brazil.

[5]Instituto Nacional de Infectologia Evandro Chagas, Fiocruz Rio de Janeiro, RJ, Brazil.

[6]Department of Clinical and Toxicological Analyses, School of Pharmaceutical Sciences, University of Sao Paulo, São Paulo, SP, Brazil.

[7]Department of Microbiology and Immunology, University of Otago, Dunedin, New Zealand.

[8]Singapore Immunology Network, Agency for Science, Technology and Research, Singapore, Singapore.

*LA: letusa.albrecht@fiocruz.br or letusaa@gmail.com

SL: stefaniecplopes@gmail.com

ABIES: iungbeatriz@gmail.com

VB: vanessa.barbosars@hotmail.com

RPA: rod.pma@gmail.com

AMS: amsiqueira@gmail.com

JAL: ju.leite84@gmail.com

NCB: najaracb@gmail.com

HGS: hellen.santos@fiocruz.br

CB: catbourgard@gmail.com

LFCG: fgtc@outlook.com

ACAVK: carolavk@gmail.com

ISS: isoares@usp.br

BR: b.russell@otago.ac.nz

LR: renia_laurent@immunol.a-star.edu.sg

ML: marcuslacerda.br@gmail.com

*FTMC: fabiotmc72@gmail.com

*Corresponding authors.

Supplementary Information

Supplementary Figure S1 - *P. vivax* rosetting frequency. Rosetting rates in 81 *P*. *vivax* isolates in the presence of autologous plasma. **(A)** Frequency distribution of rosettes among the isolates. **(B)** Boxplot showing median and interquartile ranges of rosetting rates.

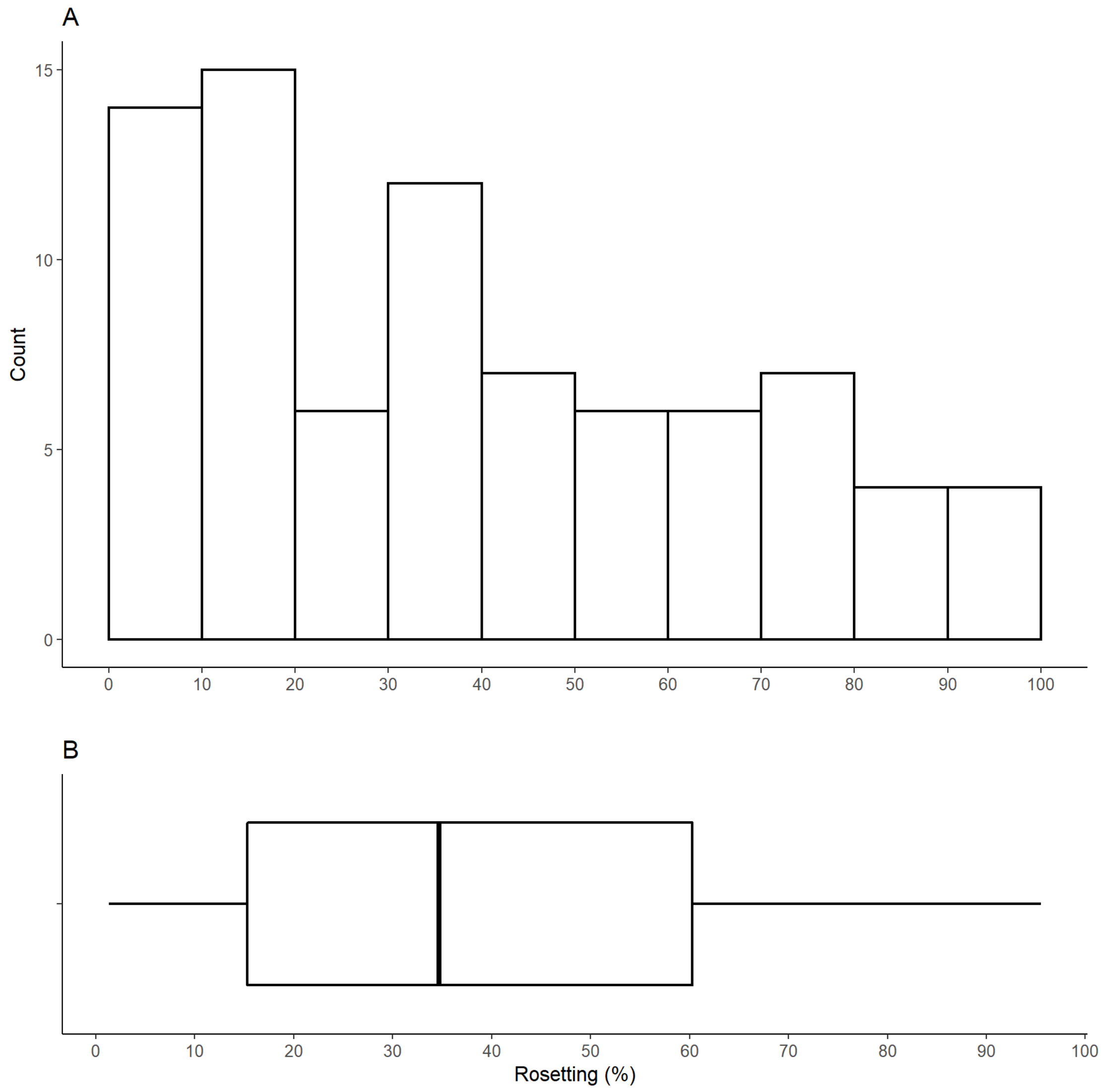

Supplementary Figure S2 - Platelet and haematocrit levels of patients infected with *P. vivax*. (A) No statistical correlation was observed when rosetting rates were compared with platelet (Pearson correlation coefficient $r = 0.09$, $p = 0.60$, n = 38) or (B) haematocrit levels (Pearson correlation coefficient $r = -0.06$, $p = 0.70$, n = 38) of patients infected with *P. vivax*.

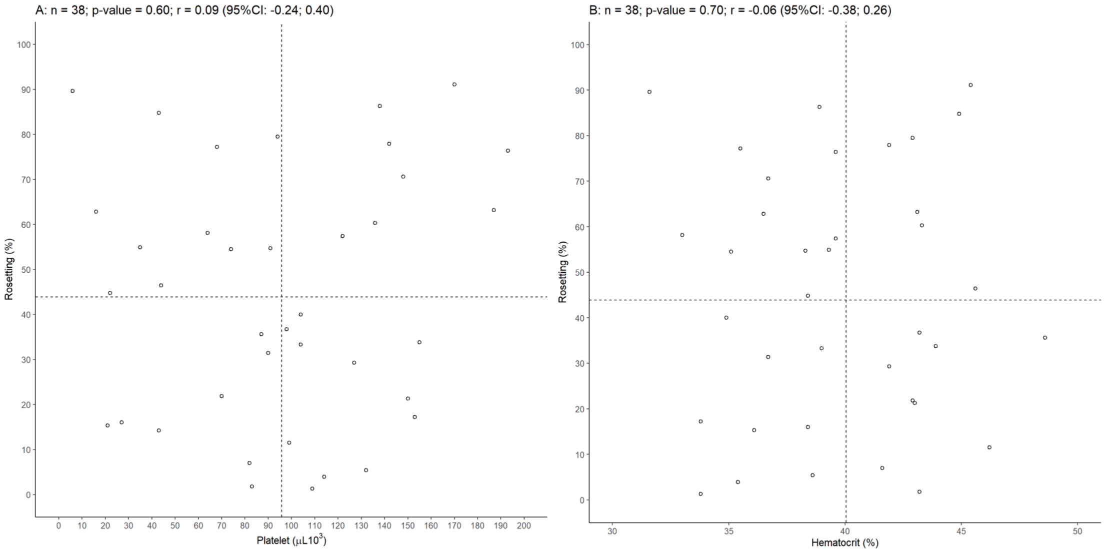

Supplementary Figure S3. (A) The binding of IgM and **(B)** IgG to red blood cells (Spearman's correlation coefficient *rho*=0.16, $p = 0.38$, n = 32, and *rho*= 0.10, $p = 0.58$, n = 35, respectively).

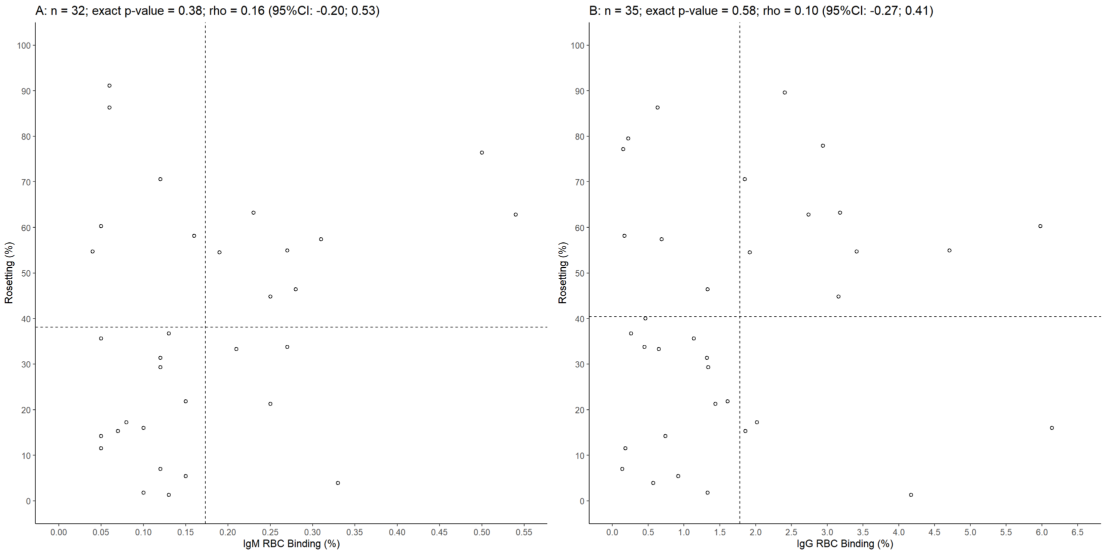

Supplementary Figure S4 - Correlation between cytokine levels and parasitemia. **(A)** Correlation between IL-6 and IL-10 plasmatic levels (Spearman's test, *rho* = 0.54, $p < 0.05$, n = 30). **(B)** Correlation between log IL-6 and parasitemia (Spearman's test, *rho* = 0.08, $p = 0.64$, n = 33). **(C)** Correlation between log IL-10 and parasitemia (Spearman's test, *rho* = 0.08, $p = 0.65$, n = 30). **(D)** Parasitemia in patients with detectable or not detectable IFN-γ (Student's t-test, $p = 0.36$ and n = 37).

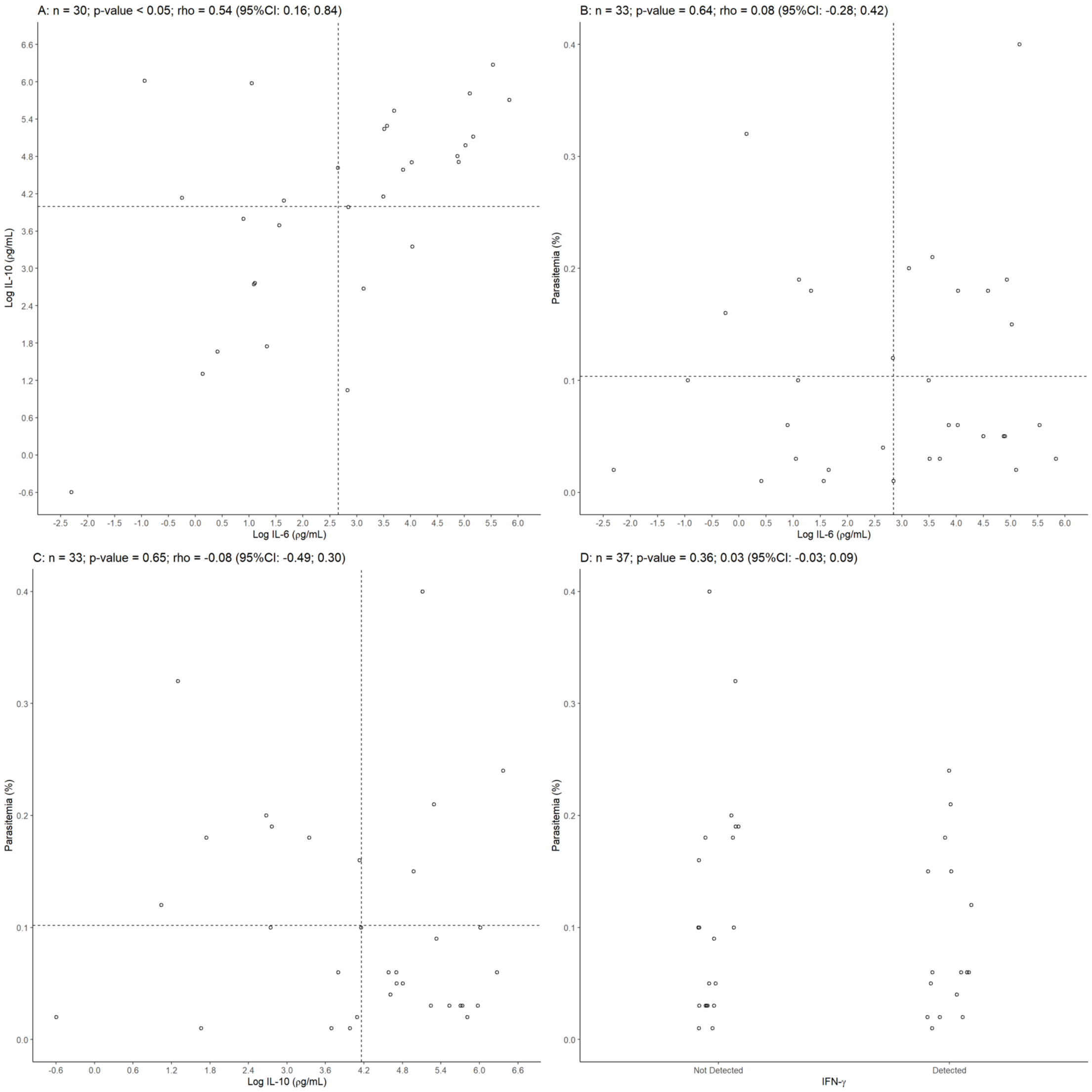

Supplementary Figure S5 - Representative images of *P. vivax* rosettes. Parasites were stained with acridine orange and visualized in optical microscopy. **(A)** Non-rosetting *P. vivax* parasite bound to only one red blood cell (RBC), not characterizing a rosette. **(B-C)** More than two non-infected RBCs binding to an infected RBC, forming a rosette.

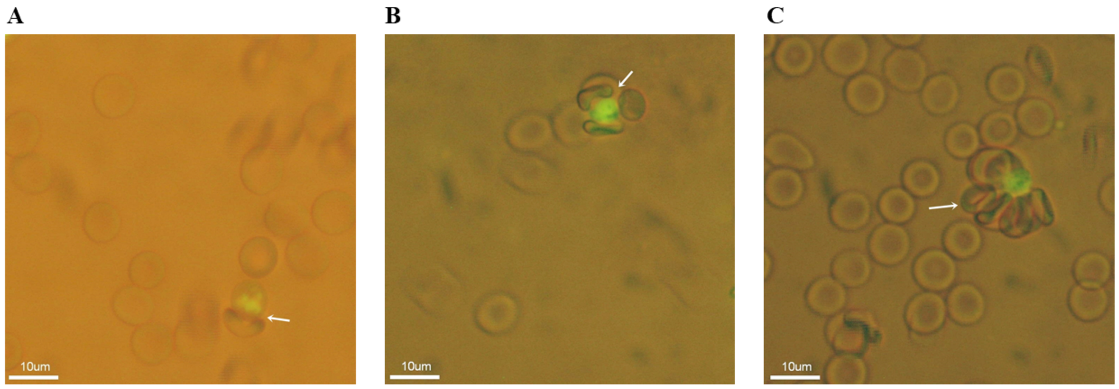

Supplement: Supplementary file 1 — Supplementary Information. [file 41598_2020_73713_MOESM1_ESM.pdf]
